# Home-based Rehabilitation With A Novel Digital Biofeedback System versus Conventional In-person Rehabilitation after Total Knee Replacement: a feasibility study

**DOI:** 10.1038/s41598-018-29668-0

**Published:** 2018-07-26

**Authors:** Fernando Dias Correia, André Nogueira, Ivo Magalhães, Joana Guimarães, Maria Moreira, Isabel Barradas, Laetitia Teixeira, José Tulha, Rosmaninho Seabra, Jorge Lains, Virgilio Bento

**Affiliations:** 1 SWORD Health, Porto, Portugal; 2 Neurology Department, Hospital de Santo António- Centro Hospitalar do Porto, Porto, Portugal; 3 Department of Population Studies, Abel Salazar Institute of Biomedical Sciences, Porto, Portugal; 4 CINTESIS, Abel Salazar Institute of Biomedical Sciences, University of Porto, Porto, Portugal; 5 EPIUnit - Instituto de Saúde Pública, Universidade do Porto, Porto, Portugal; 6 Orthopaedics Department, Hospital da Prelada- Domingos Braga da Cruz, Porto, Portugal; 7 Rovisco Pais Medical and Rehabilitation Centre, Tocha, Portugal; 8 University Institute of Maia - ISMAI, Maia, Portugal

## Abstract

In-person home-based rehabilitation and telerehabilitation can be as effective as clinic-based rehabilitation after total knee arthroplasty (TKA), but require heavy logistics and are highly dependent on human supervision. New technologies that allow independent home-based rehabilitation without constant human supervision may help solve this problem. This was a single-center, feasibility study comparing a digital biofeedback system that meets these needs against conventional in-person home-based rehabilitation after TKA over an 8-week program. Primary outcome was the change in the Timed Up and Go score between the end of the program and baseline. Fifty-nine patients completed the study (30 experimental group; 29 conventional rehabilitation). The study demonstrated a superiority of the experimental group for all outcomes. Adverse events were similar in both groups. This is the first study to demonstrate that a digital rehabilitation solution can achieve better outcomes than conventional in-person rehabilitation, while less demanding in terms of human resources.

## Introduction

With the aging population, there has been a substantial increase in the demand for Total Knee Arthroplasty (TKA). The incidence in TKA has nearly doubled^1^ from 2000 to 2013, and will continue to rise in the next decades. In the US, it has been estimated that by 2030, the demand for primary TKA will increase by 673% and for revision TKA by 601% compared to 2005^2^. This will translate into around 3.48 million primary and 268.000 revision procedures^2^.

TKA is associated with significant pain relief, functional improvements and an increase in the quality of life^3–5^. Physical rehabilitation improves results after TKA^6^, but the provision of these services varies widely in content and duration^7,8^. In the current context of increasing demand and a pressing need to contain expenditure^9^, ensuring access to effective rehabilitation while minimizing costs is both a priority and a challenge.

Currently, there are no universally accepted guidelines for rehabilitation after TKA^10,11^. There is, however, evidence favoring therapeutic exercise as the primary component^10^ and that progression upon achievement of milestones and higher intensity lead to better outcomes^12,13^, even if optimal dose and progression timings are unknown^11^.

A recent Delphi panel on rehabilitation after TKA recommended that rehabilitation should be started within 3 weeks of discharge^11^. There was no consensus on the duration, frequency, and number of treatment sessions. The greatest support was for 4 to 12 weeks of supervised rehabilitation, 2 to 3 times per week^11^.

Regarding rehabilitation setting, home and clinic-based rehabilitation protocols have generated similar improvements^14–19^. This is in line with the recent trends in healthcare delivery, towards home-based care^20^ aiming to improve cost-effectiveness.

Home-based approaches in rehabilitation, however, are very demanding both in terms of logistics and in human resources, which are scarce and costly. In an attempt to maximize access and minimize costs, tele-rehabilitation solutions have been developed and tested. For rehabilitation after knee or hip replacement, these have demonstrated similar outcomes in comparison to standard rehabilitation^21–27^, and there is preliminary evidence that these solutions may be cost-effective^28^. However, these solutions still require human supervision, either during home-based sessions or during complementary supervised sessions, limiting widespread availability.

Technological solutions that empower patients and allow home-based rehabilitation to take place without the need for real-time human supervision could be the key to improve effectiveness and lower costs. While there have been some advances in novel technologies for neurological rehabilitation, there is scant validation on solutions for musculoskeletal disorders, apart from those based on electromyography feedback^29–31^. For lower limb disorders, there is only preliminary validation of the Nintendo^®^ Wii^®^ Fit console as an adjunct to conventional physiotherapy after TKA^32,33^ or anterior cruciate ligament reconstruction^33,34^. Apart from this, we have found no validation for camera-based systems and only one study published on a solution based on inertial motion trackers^23^. This study included 142 patients, which were randomized to receive a 2-week program after surgery (10 sessions) with this system or conventional rehabilitation. The outcomes were similar in both groups, but the intervention duration was too short to draw definitive conclusions^23^.

We have tested a novel digital biofeedback system for home-based physical rehabilitation (SWORD). Using inertial motion trackers, this system digitizes patient motion and provides real-time feedback on performance through a mobile app. It also includes a web-based platform that allows the clinical team to prescribe, monitor and adapt the rehabilitation process remotely. This way, the system allows patients to perform independent rehabilitation sessions at home without the need for constant therapist supervision, ensuring remote monitoring throughout the rehabilitation program.

The present study was a single-center, parallel-group, feasibility study designed to compare the clinical outcomes of a home-based program using this system against conventional in-person home-based rehabilitation after TKA, as well as assess patient uptake and safety of this novel feedback system. We hypothesize that the clinical outcomes of such a program will be at least similar to those of traditional rehabilitation.

## Results

**Figure 1:**
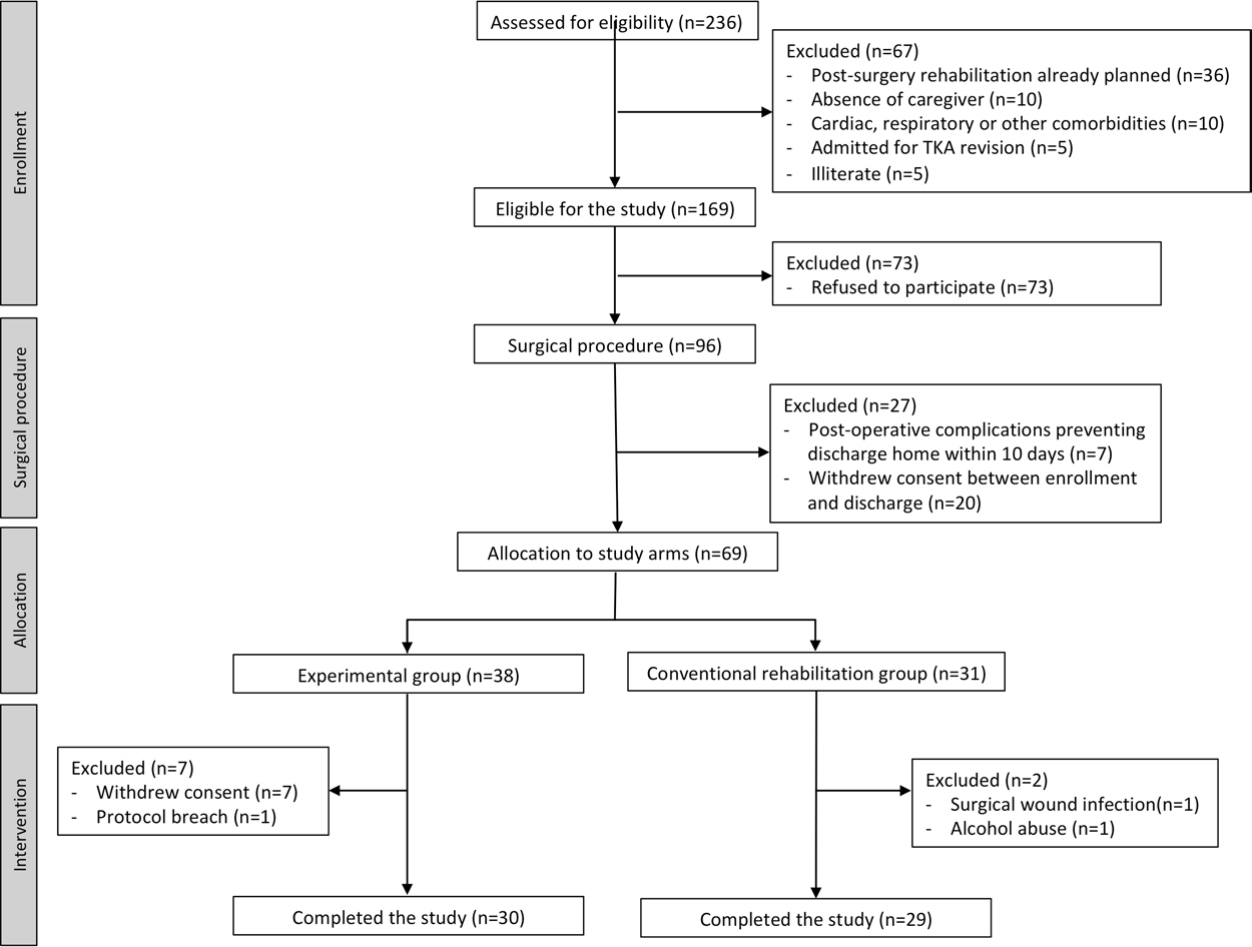
Study CONSORT diagram.

Two hundred and thirty six patients were assessed for eligibility between December 2016 and October 2017. Figure 1 shows the CONSORT diagram for the study. The study inclusion rate was of 29%. Between initial assessment and allocation to one of two study arms, a total of 93 patients refused to participate or withdrew consent, corresponding to 56% of all screening failures.

Sixty-nine patients were included and allocated to one of two study groups, according to their address of residence (37 on the experimental group and 32 on the conventional rehabilitation group). On the experimental group, 7 patients withdrew consent on the first week of the study, and one additional patient was excluded due to a protocol breach (additional physical therapy program started) corresponding to a 21% dropout rate in this group. On the conventional rehabilitation group, 2 patients were excluded, corresponding to a 7% dropout rate in this group. In total, 59 patients completed the study (30 patients in the experimental group and 29 in the conventional rehabilitation group).

### Study population characterization

Baseline characteristics of study participants regarding demographics, comorbidities and risk factors for adverse events, as well as data on hospitalization and surgery are summarized in Table 1 (total sample and divided by allocation group). There were no differences between the two study groups regarding these characteristics. The baseline assessment of the outcome variables is summarized in Table 2. At baseline, there were no differences between the two study groups regarding the primary outcome variable (TUG) nor regarding knee range of motion. Regarding the KOOS, the population in the experimental group had lower scores in every subscale.

**Table 1:** Baseline characteristics of study participants.

	Total (n = 69)	Experimental group (n = 38)	Conventional rehabilitation (n = 31)	*p* value
**Demographics**				
Age (years) mean (sd)	68.5 (7.0)	67.3 (6.8)	70.0 (7.2)	0.116^$^
Gender Female (%)	78.3	84.2	71.0	0.302^#^
Side Right (%)	55.1	63.2	45.2	0.211^#^
**Comorbidities & Known risk factors for adverse events**				
Body Mass Index mean (sd)	30.9 (4.9)	31.0 (4.5)	30.8 (5.4)	0.837^$^
Smoking (%)	11.6	10.5	12.9	1.000*
Hypertension (%)	69.6	65.8	74.2	0.623^#^
Diabetes (%)	15.9	18.4	12.9	0.743*
Pulmonary disease (%)	13.0	7.9	19.4	0.281*
Cardiac disease (%)	5.8	5.3	6.5	1.000*
Stroke (%)	0.0	0.0	0.0	NA
Renal disease (%)	1.4	0.0	3.2	0.449*
Bleeding disorders (%)	0.0	0.0	0.0	NA
ASA^€^ class 3 or 4 (%)	14.5	13.2	16.1	0.745*
Steroids for chronic condition (%)	0.0	0.0	0.0	NA
Previous contralateral knee replacement	24.6	18.4	32.3	0.296^#^
Previous hip replacement	4.3	7.9	0.0	0.247*
**Hospital admission and surgical procedure**				
Time between admission and surgery (hours)	<24 h	<24 h	<24 h	NA
Operative time (min) mean (sd)	62.6 (11.3)	62.4 (9.87)	62.8 (13.0)	0.887^$^
Minor adverse events before discharge (%)	1.4	0.0	3.2	0.449*
Hospital length of stay (days)				0.953^#^
3 or 4 days (%)	23.2	21.1	25.8	
5 days (%)	23.2	23.7	22.6	
6 days (%)	31.9	34.2	29.0	
7 to 9 days (%)	21.7	21.1	22.6	

^€^American Society of Anesthesiology physical status classification system ^#^Chi-Square test; *Fisher’s exact test; ^$^independent samples T test.

**Table 2:** Pre-operative assessment of outcome measures in study participants.

	Total (n = 69)	Experimental group (n = 38)	Conventional rehabilitation group (n = 31)	*p* value
**Primary Outcome**				
**TUG** (seconds) median (IQR)	16.59 (7.43)	18.19 (7.6)	15.98 (8.6)	0.120*
**Secondary Outcomes**				
**Range of Motion**				
mean (sd)				
Lying Knee Flexion	82.9 (16.0)	81.3 (20.2)	84.8 (31.0)	0.366*
Sitting Knee Flexion	88.0 (14.3)	85.8 (15.2)	90.7 (12.8)	0.158^#^
Standing Knee Flexion	74.8 (18.9)	72.0 (20.5)	78.3 (16.3)	0.172^#^
Sitting Knee Extension	26.2 (8.8)	27.4 (9.6)	24.8 (7.6)	0.237^#^
**KOOS**				
median (IQR)				
Symptoms	39.0 (28.0)	33.4 (14.8)	52.1 (16.4)	<0.001^#^
Pain	39.0 (16.0)	35.4 (8.7)	47.3 (12.6)	<0.001^#^
ADL	38.0 (17.0)	34.0 (25.2)	43.0 (24.0)	0.001*
Sports	0.0 (5.0)	0.0 (6.2)	5.0 (10.0)	0.004*
QOL	13.0 (19.0)	13.0 (18.0)	19.0 (31.0)	0.012*

^#^Independent samples T test; *Mann-Whitney U Test.

Considering only the patients who completed the study and that were included in the per-protocol analysis (30 patients in the experimental group and 29 in the conventional rehabilitation group), at baseline, there were no differences between the two study groups regarding TUG (p = 0.129) and knee range of motion (p = 0.345 for lying knee flexion; p = 0.187 for sitting knee flexion; p = 0.147 for standing knee flexion; p = 0.425 for sitting knee extension). Regarding the KOOS, the experimental group had lower scores in every subscale (p < 0.001 for Symptoms; p < 0.001for Pain; p = 0.005 for ADL; p = 0.006 for Sports and p = 0.007 for QOL) (see also Table 3).

**Table 3:** Outcomes assessment - change between 8-weeks assessment and baseline.

Outcome variable	Baseline assessment		8-week assessment		Change		
	Experimental group (n = 30)	Conventional rehabilitation (n = 29)	Experimental group (n = 30)	Conventional rehabilitation (n = 29)	Experimental group (n = 30)	Conventional rehabilitation (n = 29)	*p* value
**Primary outcome**							
**TUG** median (IQR)	18.2 (6.2)	15.3 (8.5)	7.8 (2.7)	10.1 (4.1)	−9.5 (8.0)	−4.6 (8.6)	0.04*
**Secondary outcomes**							
**Range of Motion**							
mean (sd)							
Lying knee Flexion	80.7 (12.4)	84.7 (18.7)	100.0 (11.3)	92.6 (13.1)	19.3 (17.0)	7.7 (16.8)	0.012^#^
Lying Knee Flexion	85.3 (16.0)	90.4 (13.1)	101.5 (9.6)	97.0 (11.3)	16.3 (17.7)	6.7 (13.5)	0.021^#^
Sitting Knee Flexion	71.6 (20.3)	18.8 (16.6)	95.8 (8.8)	86.1 (10.8)	24.2 (20.9)	7.4 (13.9)	0.001^#^
Standing Knee Flexion	26.5 (8.4)	24.8 (7.8)	14.5 (8.2)	22.8 (9.6)	−12.1 (11.1)	−1.6 (13.3)	0.002^#^
**KOOS**							
median (IQR)							
Symptoms	34.0 (20.0)	50.0 (29.0)	81.0 (14.5)	71.0 (14.0)	50.0 (26.0)	18.0 (21.0)	<0.001*
Pain	33.0 (12.0)	47.0 (24.0)	90.5 (16.0)	78.0 (16.0)	57.0 (16.3)	34.0 (25.0)	<0.001*
ADL	34.0 (18.0)	41.0 (18.0)	90.5 (0.0)	76.0 (5.0)	54.5 (16.5)	36.0 (19.0)	<0.001*
Sports	0.0 (0.0)	5.0 (8.0)	20.0 (19.0)	15.0 (19.0)	20.0 (7.5)	15.0 (15.0)	0.04*
QOL	13.0 (19.0)	25.0 (19.0)	69.0 (0.0)	56.0 (0.0)	56.0 (20.5)	31.0 (31.0)	<0.001*

^#^Independent samples T test; *Mann-Whitney U Test.

### Outcomes assessment

#### Change between baseline and the 8-week assessment

The change was superior in the experimental group in all outcome measures (see Table 3).

Based on the MCID reported in the literature for TUG (2.27 seconds)^35^ clinically significant improvements were noted in both groups. The difference between the median changes in the two groups was of 4.9 seconds, favoring the experimental group (i.e. greater improvement in this group). This difference is more than twice the MCID and therefore clinically significant.

Regarding KOOS, the improvement noted in both groups was superior to the 8–10 point MCID^36^ in every subscale, denoting clinical significant changes (see Table 3). As with TUG, the difference between the median changes in the two groups was of over 20 points in all subscales except for the Sports subscale (see a Table 3), in favor of the experimental group. Again, these differences are superior to the MCID and, hence, clinically significant.

Even though there are no MCID validated for knee range of motion in patients submitted to TKA, a study by Stratford and collaborators^37^ reported a MDC90 (Minimal Detectable Change at a 90% confidence interval) of 9.6 degrees for knee flexion and 6.3 degrees for knee extension in patients after TKA. Therefore, significant improvements in knee range of motion were noted only in the experimental group.

#### Results of the 8-week assessment

In the 8-week assessment, the TUG scores were lower in the experimental group than in the conventional rehabilitation group (median 7.8 seconds; IQR 2.7 versus 10.1 seconds; IQR 3.1) (p < 0.001), i.e. patients in the experimental group were faster than the patients in the other group.

The same was observed for knee range of motion, which was higher in experimental group for lying knee flexion (p = 0.024); standing knee flexion (p < 0.001) and for sitting knee extension (p = 0.01) but not for sitting knee flexion (p = 0.1).

Regarding KOOS, the scores in the experimental group were superior to the conventional rehabilitation group for KOOS Symptoms (p = 0.001); KOOS Pain (p < 0.001); KOOS ADL (p = 0.001) and KOOS QOL (p < 0.001), but not for KOOS Sports (p = 0.094).

#### Repeated measures analysis

This analysis was performed only for normally distributed variables - TUG and knee range of motion- after transformation. The results are summarised in Table 4.

**Table 4:** Outcomes assessment - Repeated measures analysis.

Outcome variable	Time		Group		Time*Group	
	F(df1,df2)	*p*	F(df1,df2)	*p*	F(df1,df2)	*p*
Patient performance						
TUG^#&^	F(1.7,94.1) = 104.1	<0.001	F(1,57) = 7.6	0.008	F(1.7,94.1) = 17.9	<0.001
Knee range of motion						
Lying Flexion^#&^	F(1.2,71.2) = 29.7	<0.001	F(1,57) = 0.20	0.653	F(1.2,71.2) = 4.2	0.038
Sitting Flexion^&^	F(1.3,74.6) = 23.5	<0.001	F(1,57) = 0.01	0.921	F(1.3,74.6) = 4.2	0.035
Standing Flexion^&^	F(1.3,75.1) = 30.1	<0.001	F(1,57) = 0.55	0.460	F(1.3,75.1) = 8.6	0.002
Sitting Extension^§^	F(2,114) = 14.6	<0.001	F(1,57) = 12.0	0.001	F(2,114) = 8.0	0.001

^#^ln transformation; ^§^sqrt transformation; ^&^Greenhouse-Geisser correction.

**Figure 2:**
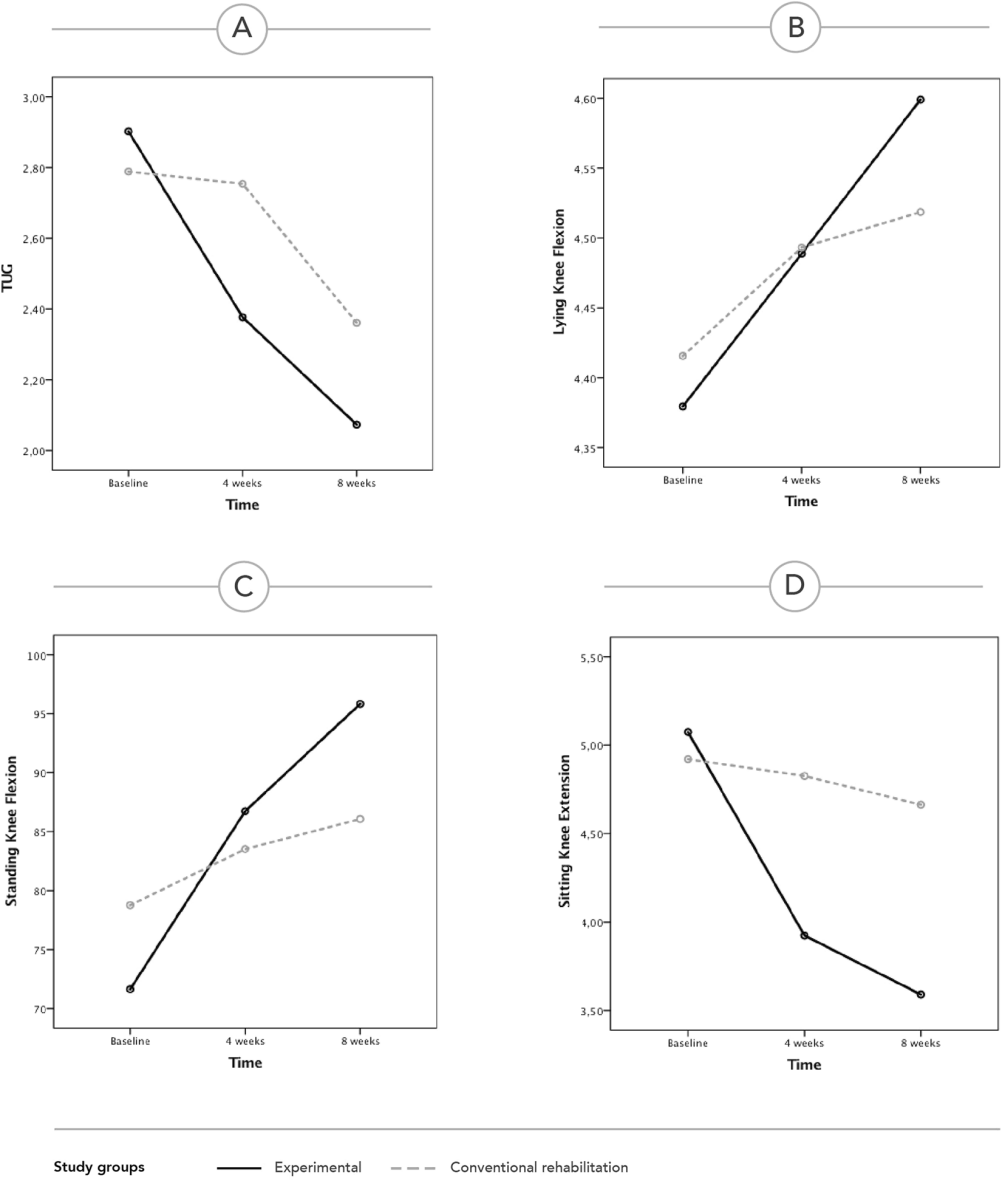
Evolution of the outcomes over time in both groups, based on the repeated measures analysis (estimated marginal means of transformed variables are presented). (**A**) TUG score. (**B**) Lying knee flexion. (**C**) Standing knee flexion. (**D**) Sitting knee extension.

While both groups presented an improvement in every dimension evaluated, this analysis revealed a main effect of time, a main effect of group and an interaction between time and group in favour of the experimental group in all outcomes (see Table 4 and Fig. 2).

### Treatment intensity

The total active treatment time was superior in the experimental group, with a median of 31.5 hours (IQR 18.0; range 10.8–69.1) versus 24 hours in the control group (p = 0.005). Time spent on additional unsupervised sessions by patients in the control group was not considered, as patients were not requested to register these sessions.

### Therapist-patient interaction

In the conventional rehabilitation group, each patient had 24 face-to-face sessions. In the experimental group, each patient had 3 face-to-face contacts with the therapist (on deployment, 4 weeks into the rehabilitation program and on termination), and on average, 0.4 (sd = 0.7; range 0–2) extra contacts for technical assistance. Regarding telephone calls, in addition to the two scheduled calls per protocol (at weeks 2 and 6), each patient received a median of 2.5 extra calls (IQR = 3.0; range 1–12).

### Independence of use

In the experimental group, 60% of the patients required the assistance of a caregiver either in motion tracker/strap placement or in the interaction with the app. There was no age difference between autonomous patients (median 65.5 years; IQR 13) or those needing assistance (median 65.5 years; IQR 13) (p = 0.185).

### Patient satisfaction

In the experimental group, patients were asked to answer the following question at the end of the program “On a scale from 0 to 10, how much would you recommend the system to one of your friends or neighbours?”. From the 30 patients, 27 (90.0%) rated the system with 10, one patient rated the system with 9 and two rated the system with 8.

### Safety and adverse events

In the experimental group, from the patients who were initially enrolled, adverse events were reported in only one patient (thrombophlebitis), corresponding to an adverse event rate of 2.6%. In the conventional rehabilitation group, from the patients who were initially enrolled, one patient was excluded due to a surgical wound infection requiring readmission to hospital and a revision procedure, and another was excluded for alcohol abuse interfering with the compliance with the rehabilitation program. Inflammatory signs over the surgical wound were reported in three additional patients and thrombophlebitis in one. This corresponds to an adverse event rate of 22.5%. This difference was not significant (p = 0.065).

## Discussion

Given the paucity of studies in this area, comparison of the results of the present study with similar studies is not possible, except with a study published by Piqueras *et al*.^23^, where a solution broadly similar to the SWORD device was tested. We will therefore consider, in addition, other studies published on rehabilitation after TKA^12,13,38^, on home versus clinic-based interventions^17^ or tele-rehabilitation versus conventional rehabilitation^23,25,26^.

In the study by Piqueras *et al*.^23^, the outcomes were similar in both groups^23^. However, in this study, the intervention time (10 sessions over 2 weeks) was too short to allow the detection of differences between groups, and treatment intensity was inferior to current recommendations^10,11^. In fact, treatment intensity is highly variable in published studies on rehabilitation after TKA and we have found only one study where treatment intensity was comparable to the one provided to the patients in the present study^12^. This study, by Bade *et al*.^12^, compared two different treatment intensities in the rehabilitation after TKA, in an outpatient setting, with the high intensity group performing 25 sessions in 12 weeks, which was similar to the treatment intensity in the conventional rehabilitation group in our study.

Previous published studies demonstrated similar outcomes for home versus clinic-based rehabilitation^12,13,17,18,38^ or tele-rehabilitation versus conventional rehabilitation^23,25,26^. The present study, however, demonstrated a clear superiority of the experimental group for all outcomes (performance tests, knee range of motion and patient reported outcomes) in terms of change between baseline and the 8-week assessment (see Table 3). Plus, for TUG and knee range of motion, this superiority was corroborated by the repeated measures analysis, which clearly demonstrates an association between these outcomes, time and intervention in favor of the experimental group (see Table 4 and Fig. 2).

Given the wide variation in terms of pre- and post- intervention TUG scores in published studies on rehabilitation after TKA^12,13,23,25,38^, comparison with published data is difficult. Still, the study by Mizner *et al*.^38^ on the time course of functional recovery after TKA reported a mean improvement of 1.7 seconds between the pre-operative and 3 months assessment, and the study by Bade *et al*.^12^ reported a mean change of 1.6 seconds in the high intensity rehabilitation group after 3 months. These results are lower than what was observed in our study for both groups. In comparison with the results reported by Petterson *et al*.^13^ in a study on progressive strengthening interventions after TKA (4.08 seconds improvement at 3 months), the results of the experimental group are superior whereas the results of the conventional group are comparable. It is however, necessary to stress that these studies reported the change between baseline and 3 months whereas we are comparing the change between baseline and 8 weeks.

Considering papers published on tele-rehabilitation solutions, the results observed in the experimental group are clearly superior to those reported by Piqueras *et al*. for the tele-rehabilitation group (−5.22 seconds change)^23^- still, with the caveat of the short timeline of the intervention in this study - but lower than those reported by Russel *et al*.^25^. However, in the latter study, the baseline TUG scores were much higher than those observed in our study (mean scores of 28.8 ± 16.6 seconds in the tele-rehabilitation group and 26.8 ± 12.1 seconds in the control group)^25^.

Regarding knee range of motion, even if these measures represent poor markers of implant success and patient satisfaction^10,39,40^, significant improvements were noted in both groups. The mean change in lying knee flexion observed in the experimental group (19.3) was comparable to that reported in other studies on home- versus clinic-based rehabilitation (15.0–17.0 degrees)^17^ and tele-rehabilitation (17.8–19.8)^25^ and higher than those reported by Piqueras *et al*. for the tele-rehabilitation group (7.7)^23^. The results in the conventional rehabilitation group were inferior to those reported by these authors, but still with a mean lying flexion angle above 90 degrees at the 8-week assessment (see Table 3). Regarding knee extension, comparison with published studies is more complicated. In this study, knee extension at baseline was much worse- 26.8 degrees (sd = 8.8) than reported by other studies^17,23,25,41^. However, we tested knee extension in a sitting position, which is more demanding than in a supine position, whereas some authors opted for the latter^12,13,38^ or simply did not specify patient position^21–23,26^.

Regarding KOOS, in the conventional rehabilitation group, the change was comparable to the change reported by Moffet *et al*. for both groups (conventional and tele-rehabilitation) regarding the Symptoms, Sports and QOL subscores (18.0 vs 14.3–16.8 points; 15.0 vs 13.1–14.3; 31.0 vs 33.9–35.6) and higher for Pain and ADL subscores (34.0 vs 23.5–26.7 points; 36.0 vs 26.0–27.2 points)^26^. In the experimental group, the median change in KOOS was significantly higher, both in comparison to the conventional rehabilitation group and in comparison to the results reported by Moffet *et al*., with differences of over 20 points in all subscales except for the Sports subscale (see above and also Table 3)^26^.

However, because the scores in the experimental group were lower in all subscales at baseline (see Tables 2 and 3) it can be argued that analyzing the change between the 8-week assessment and baseline may have benefited the SWORD group over the conventional rehabilitation group. However, the 8-week assessment also shows higher scores in the SWORD Phoenix^®^ group in all subscales except for the Sports subscale, leading to the same conclusion. Plus, the scores are also higher than the ones reported by Moffet *et al*. for the tele-rehabilitation group at the 2-month assessment, except for the Sports and QOL subscales^26^.

When analyzing these results, it is important to note that this was a feasibility study where patient allocation was performed according to geographical criteria, and not through randomization. Therefore, even if the two groups were similar in terms of demographics, comorbidities, risk factors for adverse and clinical characteristics (except for KOOS), a number of other factors (namely socio-economic) could have influenced the results.

It is also important to consider that treatment intensity was different between both groups, with the experimental group receiving more therapy hours. This is a potential confounding factor, as evidence points to a positive effect of treatment intensity on outcomes^12^. However, the difference between groups does not factor the additional unsupervised sessions that the patients in the conventional rehabilitation group were instructed to perform. Therefore, treatment intensity in the conventional rehabilitation group was likely underestimated. This is an aspect that needs to be controlled in ensuing studies. Still, even if the effect of treatment intensity on clinical outcomes is truly significant, it would mean that the digital biofeedback system enabled patients to increase treatment intensity without a corresponding increase in therapist contact time or supervision needs, which is the exact purpose of such a system.

Beyond this aspect, we hypothesize that the following factors may have played a role in the superiority of the experimental group: (a) the positive impact of a kinematic biofeedback tool on patient performance, especially regarding error correction and stimulation of a greater range of motion; (b) patient empowerment regarding their rehabilitation process; (c) high patient engagement through the use of gamification strategies; (c) the effect of remote monitoring on patient effort (that is, patients knew that their adherence and performance was being registered and monitored) and (d) the availability of objective data for clinical review, enabling data-driven decisions on program changes.

One other potential confounding factor, apart from treatment intensity, was that the rehabilitation protocols used in the study allowed a certain degree of liberty regarding the choice of specific exercises, targets, sets and repetitions. Therefore, inter-therapist variation could have influenced the results. To minimize this, all patients in the experimental group were treated by the same therapist and patients in the conventional rehabilitation group were treated by two different therapists, all equally trained and with similar levels of experience.

The inclusion rate in this study was low (29%), with a total of 93 patients refusing to participate or withdrawing consent. This corresponded to 56% of all screening failures, indicating that this, and not inclusion and exclusion criteria, was the main reason behind the low inclusion rate. In fact, the baseline characteristics of the study population (see Table 1) clearly demonstrate that the patients that were included in the study are a representative sample of a “real-world” scenario. This high refusal rate, (also observed by Piqueras *et al*.^23^) was to be expected, given that this study involved a new technological solution, which inevitably draws skepticism from the patient side, especially given the mean age of study participants (68.5 years; sd = 7.0).

From the patients who were allocated to the experimental group, 7 (18.5%) withdrew consent on the first week of the study. These patients were responsible for the difference in the dropout rates between both groups (21% vs 7%). We speculate that this may represent the difficulties of an aged population in interacting with technological systems, as is also noted by the low percentage of patients who were able to use the system autonomously (40%). This has been one of the main challenges faced by new technologies in this field, and this system is no exception. Even after many development iterations, these results demonstrate that there is still much room for improvement regarding usability by elderly patients.

Interestingly, even considering that most of the patients needed assistance in using the system, the patient satisfaction score was very high, with 90% of the patients rated the system as 10/10). When patients were asked to elaborate on the reasons for the score, almost all of them referred the ability to perform their sessions at home, whenever was more convenient, as the main reason for satisfaction. We speculate that the convenience of such a system, both for patients and their caregivers, motivates them to find strategies to overcome the difficulties associated with a new technology.

Regarding adverse events, while differences between the two groups were not statistically significant, there was a clear tendency for a greater number of patients reporting inflammatory signs over the surgical wound in the conventional rehabilitation group. We speculate that this might have been related to an underreporting of this particular adverse event in the experimental group. Being a mild situation, with no clinical relevance, and with spontaneous resolution without the need for medical attention, patients in this group may have not noticed this or neglected to report it to their physical therapist.

In conclusion, this study demonstrates that independent-home based rehabilitation after TKA with this novel digital biofeedback system is feasible, safe and effective. Based on the conclusions drawn from this study, larger, multi-centric, randomized controlled studies are now being planned, to confirm these findings. Plus, to our knowledge, this is the first study to demonstrate that a digital solution can achieve better outcomes than conventional home-based rehabilitation, while being far less demanding in terms of human resources. As such, it may represent a viable and cost-effective solution that can have a tremendous impact not only on rehabilitation after TKA but on physical rehabilitation in general, if the findings of the present study are replicated for other disorders.

## Methods

### System technical specifications

**Figure 3:**
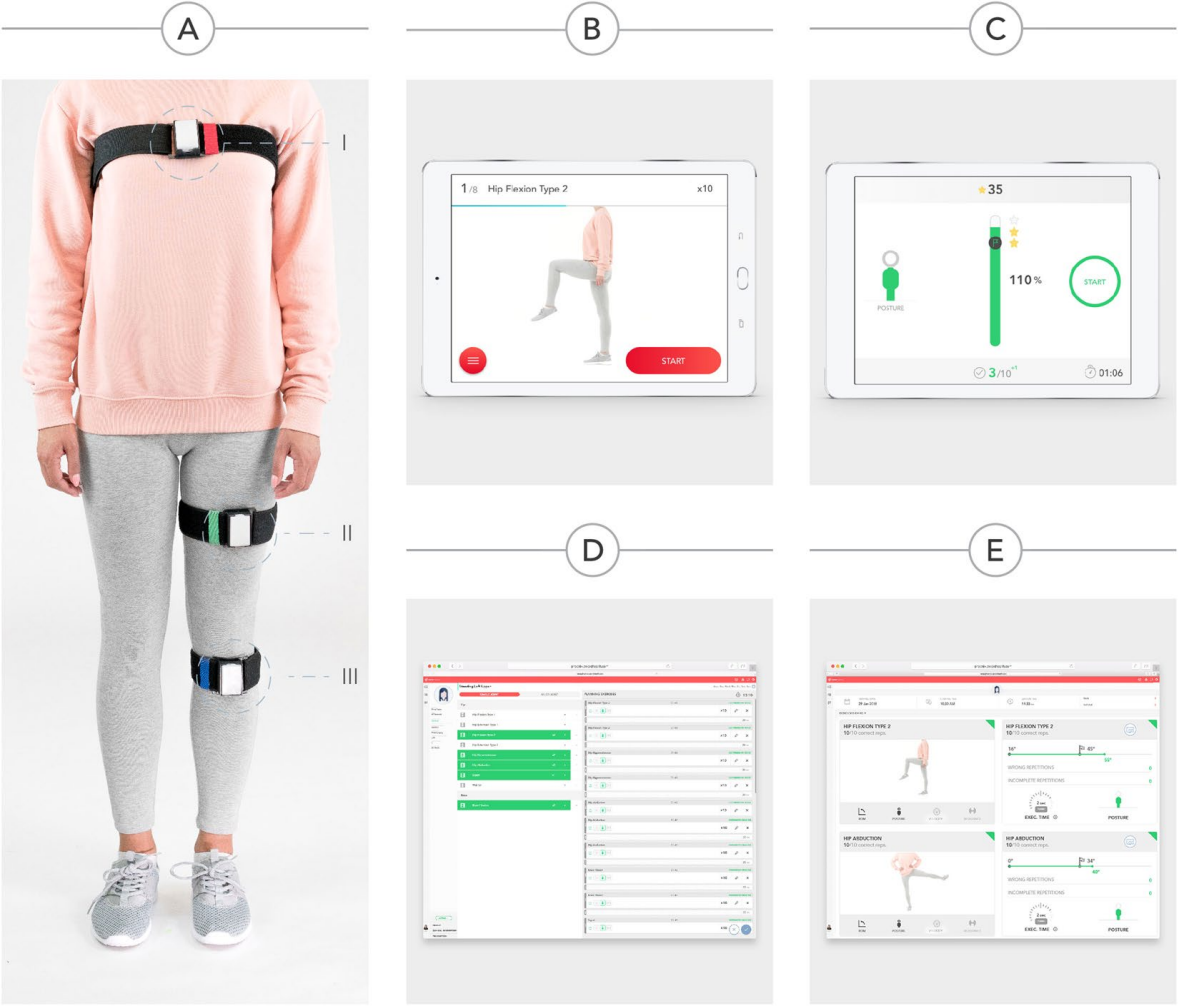
System components. (**A**) Motion Tracker Setup. (**B**) Mobile App: preparation screen. This screen is shown before each exercise, and displays a video of the exercise, as well as audio instructions. (**C**) Mobile App: execution screen. This screen is shown during exercise execution, displaying: (a) timer; (b) progress bar; (c) posture dummy; (d) repetition counter; (e) time left; (**C**) Web Portal - prescription screen. This screen displays the available exercises on the left and the layout of the exercise session on the right. (**E**) Web Portal - results screen. In this screen, the following information is presented: (a) date and time of the session; (b) session duration; (c) pain and fatigue reported by the patient through the app; (b) one card per exercise, showing baseline and target joint angles, wrong and incomplete repetitions, as well as posture errors.

The system is composed of the following components (Fig. 3A–D):

- **Inertial motion trackers (**Fig. 1A**)**
  Each tracker comprises gyroscopes, accelerometers and magnetometers, allowing 3D movement quantification. The trackers communicate via Bluetooth LE with a tablet computer. The trackers are placed on body segments using Velcro^®^ straps, in specific positions (Fig. 1A):
  I. **Red tracker:** over the sternal manubrium
  II. **Green tracker:** anterior surface of the hip, midway between the trochanter and the knee
  III. **Blue tracker:** over the anterior tibial crest
- **Mobile App**
  Before each exercise, a video demonstration is presented to the patient (Fig. 3B), complemented with an audio explanation. During execution, the patient is given real-time biofeedback through a dedicated interface (Fig. 3C). Only repetitions assessed as correct contribute to reach the session´s goals. These are defined as movements starting at the baseline and reaching or surpassing the specified range of motion without violating movement or posture constraints. If the patient violates a constraint, an error message is displayed, allowing the patient to correct the movement in the following attempts.
- **Web-based Portal**

The Portal allows clinical teams to prescribe exercises, monitor results and edit prescriptions. To prescribe a session, the clinician selects the exercises, number of sets, number of repetitions and range of motion for each exercise **(**Fig. 3D**)**. Upon the performance of a session, the results are uploaded to the platform and available for review **(**Fig. 3E**)**. Based on this information, the clinical team can edit the session remotely.

### Primary outcome

The primary endpoint was the change in patient performance at the end of the 8 week rehabilitation period compared to the baseline, measured through the Timed Up and Go Test (TUG).

### Secondary outcomes

The secondary endpoints were the change at the end of the 8 week rehabilitation period compared to the baseline regarding: a) patient reported outcomes, measured by the Knee Osteoarthritis Outcome Scale (KOOS); b) knee range of motion in degrees in the following exercises- lying, sitting and standing knee flexion; sitting knee extension.

### Location

Patients were recruited at Hospital da Prelada - Dr. Domingos Braga da Cruz, Porto, Portugal

### Sample size estimation

Sample size estimation was performed considering the primary outcome measure - TUG - in an equivalence scenario, based on the study published by Mizner *et al*.^38^ (baseline TUG sd = 2.4 seconds), where patients performed a rehabilitation protocol broadly comparable to the one used in the present study. A Minimal Clinically Important Difference (MCID) change of 2.27 seconds was considered, based on the study published by Yuksel *et al*.^35^ Considering a power of 90%, a two-sided 0.05 significance level and a 15% dropout rate, 55 patients would be necessary to detect a 2.27 second difference between the two groups. Given the wide variation in the standard deviation of the TUG reported by different authors - from 0,5 seconds^42^ to 6,3 seconds^23^- we decided to increase sample size to 70 patients, to account for a greater variation than the one reported by Mizner and collaborators.

### Participants

Patients admitted for TKA between December 19^th^ 2016 and October 16^th^ 2017 were screened for eligibility.

Subjects were included if they were ≥ 18 years old and had: (a) clinical and imaging evidence of knee osteoarthritis; (b) indication for TKA according to the patient´s orthopedic surgeon; (c) ability to walk unaided, with unilateral or bilateral support; (d) availability of a caregiver to assist the patient after surgery.

Exclusion criteria were: (a) admitted for revision of TKA; (b) contralateral hip or knee osteoarthritis severely limiting patient mobility and ability to comply with a rehabilitation program; (c) aphasia, dementia or psychiatric comorbidity interfering with communication or compliance to the rehabilitation process; (d) respiratory, cardiac, metabolic or other condition incompatible with at least 30 minutes of light to moderate physical activity; (e) major medical complications occurring after surgery that prevented the discharge of the patient within 10 days after the surgery; (f) other medical and/or surgical complications that prevent the patient from complying with a rehabilitation program; (g) blind and/or illiterate patients.

### Patient allocation

Patient allocation was performed using patient address as criterion. Subjects residing in areas outside the administrative limits of the city of Oporto were allocated to the experimental group. Conversely, patients residing within the administrative limits of the city were allocated to the conventional rehabilitation group.

### Blinding

The nature of the study did not allow blinding of the patients. Patient assessment was performed by one investigator- J.T. - blinded for study groups. Statistical analysis was performed by a blinded statistician - L.T.

### Patient assessment

Several studies suggest that the outcomes should be measured not only in terms of range of motion, a poor marker of implant success and patient satisfaction^10,39,40^ but also using patient-reported outcomes and a performance test^43,44^.

The performance test chosen was the TUG^45^, which was validated for patients submitted to TKA by Yuksel *et al*.^35^. The TUG consists on the time that a person takes to rise from a chair, walk three meters, turn around, walk back to the chair, and sit down. For patient reported outcomes, the KOOS scale^46^, which was validated for patients submitted to TKA by Alviar *et al*.^47^, was chosen. The KOOS consists of 5 subscales: (1) Pain; (2) other Symptoms; (3) Function in daily living (ADL); (4) Function in sport and recreation (Sport/Rec) and (5) knee related Quality of life (QoL). The previous week is the time period considered when answering the questions. Standardized options are given (5 Likert boxes) and each question is assigned a score from 0 to 4. A normalized score (100 indicating no symptoms and 0 indicating extreme symptoms) is calculated for each subscale.

Patients were assessed at baseline (pre-operatively), 4 weeks after initiation of rehabilitation and at the end of the program. Data was collected on: (a) demographics (gender, date of birth); (b) affected side; (c) comorbidities and risk factors for adverse events^48^; (d) TUG; (e) KOOS and (f) active knee range of motion (lying, sitting and standing knee flexion; sitting knee extension) measured automatically by the SWORD device.

### Safety and adverse events

Patients in the conventional rehabilitation group were under regular monitoring by a physical therapist, enabling early detection and reporting of adverse events. In the experimental group, safety was evaluated through pain and fatigue scores (graduated from 0 to 10) at the end of each session. These were available for remote monitoring. Patients were also asked to report any adverse events to their physical therapist through a direct telephone contact.

### Intervention

Rehabilitation protocols (see Table 5) were designed based on a recent systematic review^10^, the results of a Delphi panel on best practices for rehabilitation after TKA^11^ and the protocols published by SOFMER, the French Physical and Rehabilitation Medicine Society^49^.

**Table 5:** Rehabilitation protocols used in the study.

Stage	Weeks	Experimental group	Conventional rehabilitation
1	0–2	—	Soft tissue massage
		—	Active assisted mobilization of the knee to increase range of motion
		—	Gait training with bilateral support
		Open kinetic chain exercises without added resistance: lying, sitting and standing (with support)	Open kinetic chain exercises without added resistance
		Strengthening of hip flexors and extensors	Strengthening of hip flexors and extensors
		Ice pack application after each session and throughout the day as needed	Ice pack application after each session and throughout the day as needed
2	3–6	—	Soft tissue massage
		Exercises with steps	Active assisted mobilization of the knee to increase range of motion
		Open kinetic chain exercises with added resistance, progressing to closed kinetic chain exercises, with strengthening of knee flexors/extensors and knee stabilization	Open kinetic chain exercises with added resistance, progressing to closed kinetic chain exercises, with strengthening of knee flexors/extensors and knee stabilization
		Progression to standing exercises without support	Gait training with progressive withdrawal of external support
		Ice pack application after each session and throughout the day as needed	Ice pack application after each session and throughout the day as needed
4	7–8	Eccentric strengthening exercises	Eccentric strengthening exercises
		Exercises involving steps	Exercises involving steps
		Multi-directional exercises	Weight-bearing exercises
		Ice pack application after each session	Ice pack application after each session

Both groups received home-based rehabilitation for 8 weeks starting between day 7 and day 10 after surgery.

The experimental group performed a rehabilitation program solely through the use of the biofeedback system. After an initial deploy and training visit, the program was monitored remotely by the assigned physical therapist. Patients were instructed to perform exercise sessions between five and seven days a week, but were not excluded from the study in case of lower adherence. Total training time was registered automatically by the device. Each patient received a visit from the physical therapist 4 weeks after initiation of the program and then a termination visit. Two interim telephone calls were also scheduled (at 2 and 6 weeks after initiation of the rehabilitation program). Additional telephone or face-to-face visits were performed when required and registered.

The conventional rehabilitation group received a program provided by a physiotherapist, 3 times a week, for 1 hour. Patients were also instructed to perform additional unsupervised sessions in at least two other days of the week. Compliance to these additional sessions was not mandatory.

### Statistical analysis

To assess differences in clinical and demographic variables of the patients allocated to the two study groups, independent samples t test or Mann–Whitney U test were used for quantitative variables. For qualitative variables, Chi-squared test or Fisher’s exact test were used.

Outcome analysis was performed using a per-protocol analysis. The impact of the interventions in the primary and secondary outcomes was evaluated considering the change between baseline and week 8. Differences between the two study groups were performed using independent samples t test or Mann-Whitney U test. Since outcomes were measured in three different moments (baseline, 4 weeks and 8 weeks), a repeated measures analysis was also performed, using a 3 × 2 ANOVA with group as an independent factor and time as a within- subjects factor. When necessary, logarithm or square root transformations were performed to obtain normally distributed variables. In all analysis, a significant level of 0.05 was considered.

### Ethics approval of research

The study was approved by the National Data Protection Commission (authorization number 1476/2017) and by the local ethics committee at Hospital da Prelada (Chair: Dr. Juiz Conselheiro Almeida Lopes). The methods were conducted in accordance with the approved guidelines. All patients and caregivers were provided with information about the purpose and procedures of the study and provided written informed consent before inclusion. All patient data was anonimized and linked to the patient by a unique study number that did not contain any personal identifiers.

### Clinical Trial Registration

This clinical trial was prospectively registered at www.clinicaltrials.gov with the Unique identifier: NCT03047252. Date of registration: 8 February 2017.

### Availability of data and materials

The study protocol is available from www.clinicaltrials.gov. Individual patient data that underlie the results reported in this article was submitted as supplementary information (see Supplementary Dataset 1) which can be accessed through the online version of this paper.

## Electronic supplementary material

Dataset 1
